# Decreasing Abundance, Increasing Diversity and Changing Structure of the Wild Bee Community (Hymenoptera: Anthophila) along an Urbanization Gradient

**DOI:** 10.1371/journal.pone.0104679

**Published:** 2014-08-13

**Authors:** Laura Fortel, Mickaël Henry, Laurent Guilbaud, Anne Laure Guirao, Michael Kuhlmann, Hugues Mouret, Orianne Rollin, Bernard E. Vaissière

**Affiliations:** 1 INRA, UR 406 Abeilles et Environnement, Avignon, France; 2 UMT Protection des Abeilles dans l'Environnement, Avignon, France; 3 Department of Life Sciences, Natural History Museum, London, United Kingdom; 4 Arthropologia, Ecocentre du Lyonnais, La Tour de Salvagny, France; 5 ACTA, Site Agroparc, Avignon, France; University of Guelph, Canada

## Abstract

**Background:**

Wild bees are important pollinators that have declined in diversity and abundance during the last decades. Habitat destruction and fragmentation associated with urbanization are reported as part of the main causes of this decline. Urbanization involves dramatic changes of the landscape, increasing the proportion of impervious surface while decreasing that of green areas. Few studies have investigated the effects of urbanization on bee communities. We assessed changes in the abundance, species richness, and composition of wild bee community along an urbanization gradient.

**Methodology/Principal Findings:**

Over two years and on a monthly basis, bees were sampled with colored pan traps and insect nets at 24 sites located along an urbanization gradient. Landscape structure within three different radii was measured at each study site. We captured 291 wild bee species. The abundance of wild bees was negatively correlated with the proportion of impervious surface, while species richness reached a maximum at an intermediate (50%) proportion of impervious surface. The structure of the community changed along the urbanization gradient with more parasitic species in sites with an intermediate proportion of impervious surface. There were also greater numbers of cavity-nesting species and long-tongued species in sites with intermediate or higher proportion of impervious surface. However, urbanization had no effect on the occurrence of species depending on their social behavior or body size.

**Conclusions/Significance:**

We found nearly a third of the wild bee fauna known from France in our study sites. Indeed, urban areas supported a diverse bee community, but sites with an intermediate level of urbanization were the most speciose ones, including greater proportion of parasitic species. The presence of a diverse array of bee species even in the most urbanized area makes these pollinators worthy of being a flagship group to raise the awareness of urban citizens about biodiversity.

## Introduction

Urbanization is one of the main human activities that causes drastic and irreversible habitat alterations, and it is likely to increase in the coming years [1]. Urban environments are defined as mosaics of impervious and permeable surfaces that harbor regularly disturbed habitats [2]. In urbanized landscapes, green areas decrease with a corresponding increase of impervious surface, which includes buildings, roads and industrial areas. An urban environment can thus be characterized by its proportion of impervious surface and the level of connectivity among its patches of permeable surface, both of which have an impact on the fauna [3]–[5].

Even if urbanization has negative impacts on the insect fauna [6]–[9], many bee species are common within urban areas [3], [8]–[11]. Indeed, man-made environments like urban habitats and gardens can host a rich and abundant wild bee fauna [12]–[14]. For example, 262 bee species were recorded within the city of Berlin, Germany, over five years [9]. Matteson *et al*. (2008) collected 54 bee species in 19 urban gardens, and Fetridge *et al*. (2008) recorded 110 species in 21 residential gardens, both studies were conducted over two years in New York City during the summer months [13], [15]. For a bee species to be present in a given habitat, it must be able to find food and nesting substrate within its species specific range of activity [16]. Urban and periurban sites can provide high quantities of flowers all year long [15], they show a high diversity of land-cover types, and are often warmer than surrounding landscapes [17]. Also, such habitats are seldom treated with pesticides [10] which are involved in the decline of bees elsewhere [18].

Williams *et al*. (2010) demonstrated that ecological traits can be used to predict bee responses to a variety of disturbance types [19]. Indeed, the presence of a bee species may be jeopardized by the fragmented nature of urban habitats because of its limited flight ability. Concerning the nesting behavior, some bees are soil-nesting, while others nest above ground in stems, dead wood or walls (cavity-nesting species). The regular disturbance in urban habitats (e.g. mowing, weeding or soil plowing) may prevent the long-term establishment of soil-nesting bee species [13], which represent over 80% of the bee fauna worldwide [20]. There is also some evidence that cavity-nesting species are over-represented in urban bee communities [3], defined as the assemblage of species populations that occur together in space and time [21]. Every species has its own functional traits and will respond accordingly to habitat alteration that characterizes urban environments [22]. Therefore, the species and its functional traits are essential elements to study the impact of urbanization on wild bee community structure, defined as the species diversity found in a given area. Indeed, several studies have documented the changes in wild bee community structure in urban environments [6], [23], [24].

It is unknown whether, and if so how, the proportion of impervious surface and the level of connectivity among permeable surfaces combine to affect the structure of wild bee communities. Only few studies have surveyed bee communities along a gradient of urbanization [23], [24]. In most cases, the effect of urbanization on bee communities was analyzed using different categories of landscapes such as urban, periurban or natural areas [6], [13], [15]. We did not choose this approach, but rather we followed McDonnell and Hahs (2008) and McDonnell and Pickett (1990) and used a gradient to assess the effects of urbanization [25], [26]. Our objectives were to 1) assess the wild bee community structure along an urbanization gradient; 2) test the effects of the proportion of impervious surface and the level of connectivity among permeable surfaces on the wild bee abundance and species richness; and 3) investigate the changes of composition in the wild bee community along the gradient in relation to functional traits.

## Materials and Methods

### Study sites

The study was conducted in the urban community of Grand Lyon, France, which includes 58 towns around Lyon (45° 46′N, 4° 50′E) and covers an area of 516 km^2^. With approximately 1.3 million inhabitants [27], this urban community consists of diverse ecosystems ranging from densely populated urban areas to intensive agricultural landscapes or semi-natural grasslands. The climate of Lyon is at the temperate-Mediterranean interface. Located in the Rhône valley, the wind commonly blows from the south. The 30-year annual average temperature is 12°C with a minimum of 3°C in January and a maximum of 21°C in July [28].

**Figure 1:**
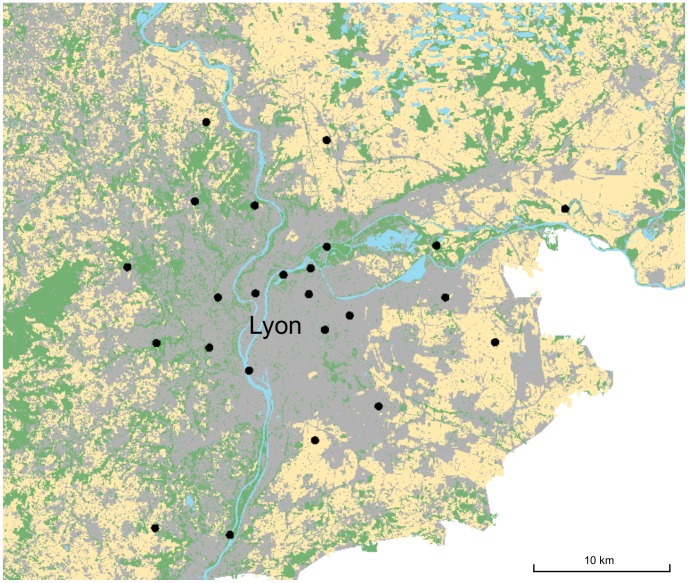
Distribution of the 24 sites along the urbanization gradient around Lyon, France. Base map colors represent: impervious surface (grey), agricultural landscape (yellow), semi-natural habitat (green) or water (blue).

We selected twenty-four sites following a increasing gradient of impervious surface (from 10 to 95%) over a two kilometer radius in different directions from the downtown Lyon area (Figure 1), and secured appropriate authorizations from the different authorities for each of them (farmer, city,…; see Table S1). Thus, eight sites were covered by less than 30% of impervious surface, eight by a proportion between 30 and 70%, and the remaining eight by more than 70% of impervious surface. For part of the surveys, we captured bees on flowers, so sites were chosen in green areas, parks or gardens. All sites were distant by more than two kilometers from each other to prevent overlapping bee communities [29].

### Wild bee surveys

We used both pan traps and insect nets to assess the bee community at each site in 2011 and 2012 [30], [31]. Pan trapping is a standard method for catching bees [30], though it is known to perform poorly for some taxa [32]. It is a passive method based on the visual attraction to colored pan traps and it provides quantitative data on the abundance of a large part of the wild bee fauna without the bias associated with the difference in capture efficiency among observers using active collecting methods (e.g. netting) [30], [33]–[36]. We used 500 ml plastic bowls painted with yellow, blue or white fluorescent paint (Rocol Top, France) [30], [31]. Pan traps were arranged in triplets, with each triplet consisting of a pan of each of the three colors randomly distributed either at the corners of a three meters side equilateral triangle, or, when space did not permit otherwise, linearly with three meters between two adjacent bowls. The pan traps were set at a height slightly above that of the average vegetation, and they were activated by filling them with 400 ml of water with a drop of detergent, and left active for 24 hours. Pan trapping is very sensitive to the immediate environment [37]. In order to take this effect into account, we set two triplets of pan traps separated by 20 to 40 m from each other [38], one being in an open area and the other along the sunniest side of a vertical landscape element (edge, wall, or tree). From March until October, we sampled bees on the same day for all 24 sites on a monthly basis.

Net surveys were done from March until September on a monthly basis also right after pan trapping by a range of observers so that it lasted between five and eight days (weather did not permit to do these observations in October in both years). At each study site, we surveyed all flowering plant species in bloom within a radius of 100 m around the centroid of pan traps, except for grasses since we found no records of wild bees foraging on flowers in the Poaceae family in Europe. For each species, flowers were observed for up to two minutes. Observation then stopped if no foraging activity was detected. Else, the first bee observed was caught and net catching lasted for five minutes after this first capture. Sampling took place alternately in the morning and in the afternoon at each site to cover the whole foraging bee population [39].

The Plant diversity was recorded for each site in April and July 2012, over two perpendicular transects of 50 m each centered on the centroid of the pan trap triplets. One transect was aligned along the centers of the two pan trap triplets and the other one was perpendicular. At each date, all plants (in bloom or not) on these transects were identified to species by professional botanists. In that way, we had a standardized and exhaustive estimation of the plant diversity of each site.

Pan trapping and net sampling were performed only during periods of good weather for foraging activity (maximum temperature ≥15°C, sunny sky or with scattered clouds only, and wind speed ≤15 km/h [40]). Specimens collected in pan traps were first stored in 70% ethanol (w/w) until washed and dried following Lebuhn (2013). All these specimens as well as sweep samples were frozen for later processing. Individuals were then pinned, labeled, and sent for identification to species to the respective authority for each genus (see Acknowledgements). All voucher specimens are now deposited in the bee collection of INRA Avignon. For taxonomy, we followed the nomenclature of Kuhlmann *et al*. [41] (see Table S2 for the entire species list). Honey bees (*Apis mellifera*) were caught in pan traps and observed during net sampling, but they were not considered in this study so that ‘bees’ will be used synonymously with ‘wild bees’ in the following unless stated otherwise.

### Landscape structure

To characterize the landscape surrounding each study site, we used the Geographic Information System Arcgis v 9.3 and Fragstat software [42]. Landscape characteristics were analyzed at the three radii of 500 m, 1000 m, and 2000 m centered on the centroid of the two pan-trap triplets. These radii were chosen because flight distance of wild bees are estimated between a few hundred meters to several kilometers depending on the species [43]–[49]. The minimum size of habitat patches was defined by the spatial resolution of our raster, which was of 256 m^2^ (i.e. 16 m×16 m). We used seven mutually exclusive land-cover types: roads, buildings, industrial areas, agricultural land, wooded areas (e.g. forests, hedgerows), open areas (e.g. meadows, bare soils areas), and water. Based on principal component analyses of the proportion of land-cover types at each site, the proportion of roads, buildings, and industrial areas were strongly correlated with the first axis (see Figure S1). These three variables were therefore pooled together as the proportion of impervious surface (Impervious surface) for further analyses. There was a clear gradient in the proportion of impervious surface among the sites that ranged between 0–98%, 1–98%, and 12–93% at the radii of 500 m, 1000 m, 2000 m, respectively. In addition to land-cover uses, we calculated the variables Connectivity of open area and Connectivity of wooded area. Landscape connectivity is defined as the degree to which the landscape facilitates or impedes movements among resource patches [50]. In this study, connectivity is defined as the number of functional joinings between patches of the same type, where each pair of patches is either connected or not, based on a user specified distance criterion (here 100 m, that is the radius surveyed for net captures) [42]. Connectivity is the percentage of patches of a given land-cover distant from each other by a maximum of 100 m (connectivity = 100 when all patches in the landscape are connected; [42]).

### Data analyses

Bee community parameters were computed separately for each of the two consecutive years. Species diversity was characterized by species richness (using EstimateS v 9.1.0 [51]) and rank abundance distribution (using BiodiversityR package in R v 2.15.2 software [52], [53]). The observed cumulative species richness curve and the total expected species richness were computed using a bootstrapping procedure with 1000 random reorganizations of sampling order. Total expected species richness was assessed using the Jack1 and the Chao2 estimators because they are the least biased estimators for species-rich assemblages [54]. The proportions of singletons (species represented by a single specimen) and of species for each modality of the functional traits were further compared for each year by means of Chi-square tests.

Pearson correlation coefficients were calculated to quantify how the landscape variables were correlated with each other (see Table S3 for further information). When variables were significantly correlated with Impervious surface, we kept only this latter variable for final analyses. Because of the high correlation between the measurements at the three radii (*p*<0.001), the analyses were performed separately for each radius. After correlation analyses, we examined the effect of landscape variables on bee richness and abundance using generalized linear models (GLM). Pan-trapping data were used to analyze abundance and data from both sampling methods were used to analyze species richness and composition [30]. Normality of the abundance and richness data was tested by Shapiro tests. As abundance data were skewed to the right, a log-transformation was performed to normalize data before analyses. At each radius, models were simplified by forward selection based on AIC (Akaike Information Criterion) values. We then considered the model with the lowest AIC value as the most parsimonious one.

To further determine which ecological processes would best explain changes in species composition along the urbanization gradient, we performed complementary analyses that incorporated species-specific information on functional traits [19], [55]. We first compared the response of parasitic vs. non-parasitic species to landscape variables. Then, for non-parasitic species, we gathered information on tongue length, nesting behavior, and social behavior from published information [20], [56]–[61]. Pollen diet specialization will be analyzed elsewhere in relation with the composition of the local flora. Species of the families Apidae and Megachilidae were considered as long-tongued and the others as short-tongued. Species were divided into the following binary ecological categories: soil-nesting or cavity-nesting for the nesting behavior, and solitary (each female constructs her own nest and provides food for her offspring) or social (from gregarious to eusocial) for social behavior [20], [62]. We also used body size by measuring the inter-tegular distance (ITD) with a dissecting microscope and calibrated ocular micrometer on a sample of 3 to 10 randomly selected female specimens per species. The ITD measures the width of the thorax, which contains the flight muscles, and it is related to dry body mass and also to foraging distance [44], [63]. A total of 58 species could not be included in these analyses due to partly missing information on functional traits. GLMs were performed on the occurrence frequency of bee species in all sites based on landscape variables in interaction with functional traits. In all GLMs, the effect of each landscape variable was nested in the year to account for interannual variations.

Whenever a large number of different tests are conducted, one uses a correction for multiple comparisons (often the Bonferroni adjustment [64]) because series of non-independent tests increase the probability of significant results due to chance only. Thus, we used a three-fold Bonferroni correction for abundance and richness analyses repeated throughout the three spatial scales and a five-fold correction for species occurrence analyses repeated along the five functional trait categories.

## Results

### Characterization of the bee fauna

**Figure 2:**
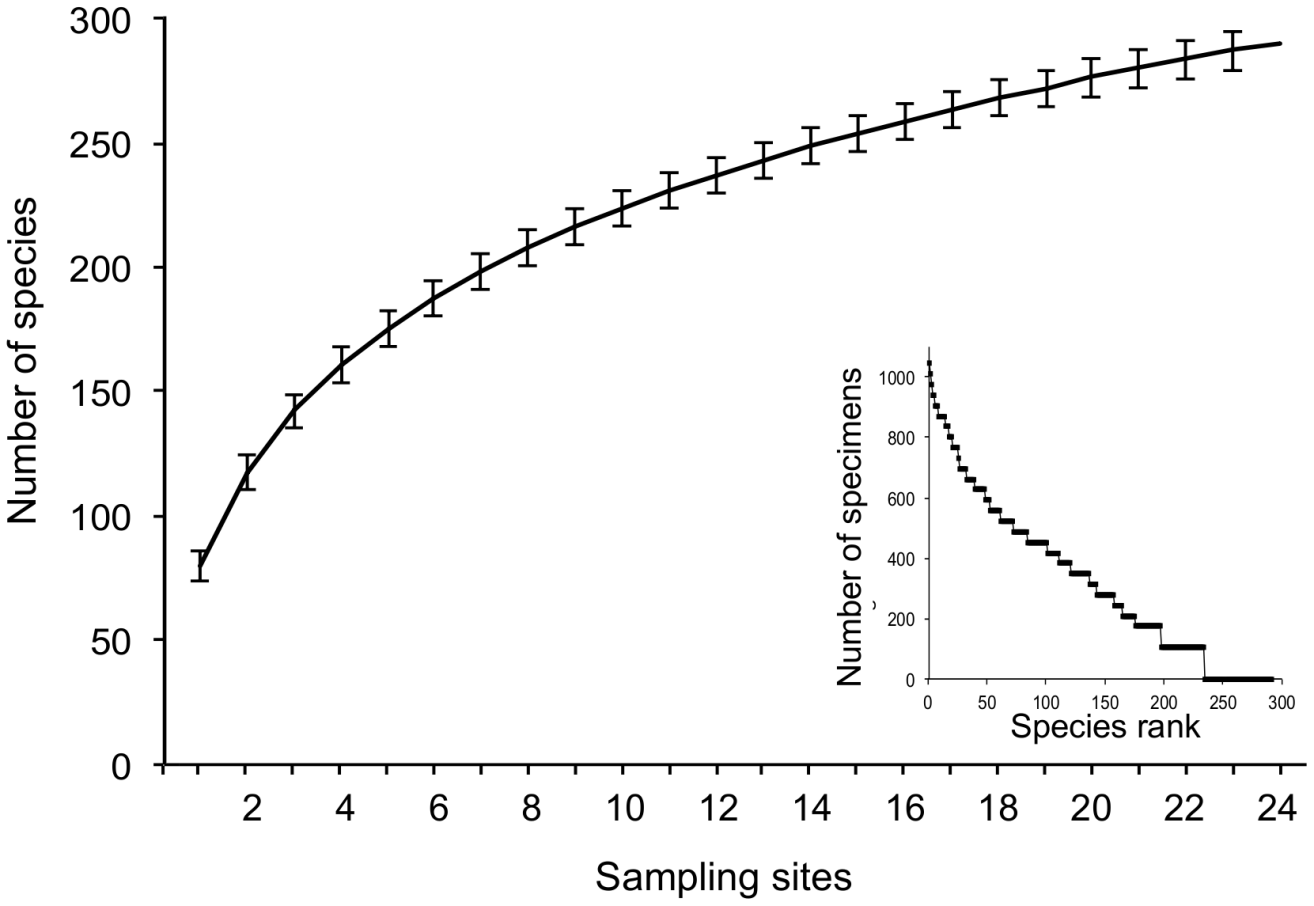
Mean species accumulation curve for pooled data from 2011 and 2012 (1000 randomizations).

Over the two years of survey, a total of 12872 bee specimens were collected, 7187 in 2011 and 5685 in 2012. They belonged to six families (Andrenidae, Apidae, Colletidae, Halictidae, Megachilidae, Melittidae), 34 genera and 291 species (256 in 2011 and 226 in 2012). Halictidae had the largest diversity with 59 different species, while there were only two species in the Melittidae. A total of 100 species were collected only in one of the two years (65 in 2011 and 35 in 2012), which represents 34% of the recorded species. Species accumulation curves did not reach saturation, which indicates that we did not capture all the species potentially present in our study area (Figure 2). Using EstimateS, the predictor of estimated species richness over both years pooled together was 366.7 for Chao2 and 367.7 for Jack1 (Table 1). Thus nearly 79% of the estimated number of bee species present in the study area were recorded for the two methods combined over the two years.

**Table 1:** Observed and estimated species richness.

Year	Sobs*±SD**	Chao 2±SD (completeness)	Jack 1±SD (completeness)
2011–2012	291±7.87	366.71±22.49 (79.35)	367.67±11.56 (79.15)
2011	256±8.43	350.09±28.23 (73.12)	333.63±11.32 (76.73)
2012	226±7.96	309.95±26.51 (72.91)	295.96±11.2 (76.36)

*Sobs = observed species richness.

**SD = standard deviation.

The proportion of singletons was not significantly different between the two years (*χ* ^2^ = 1.26, *df* = 1, *p* = 0.26), nor were the proportions of species among each modality of the functional traits (*χ* ^2^≤0.69, *df* = 1, *p*≥0.4). Overall, 57 species (20% of the total) were recorded as singletons and 37 (13%) as doubletons. Among singletons, 11 species (19.5%) were parasitic and among all species, there were 49 parasitic ones (17%) and 242 non-parasitic ones. Non-parasitic species were dominated by solitary species (74%), short-tongued species (67%) and soil-nesting species (69%). Twenty-two species represented each from 1% to 4% of the total number of specimens (138 to 565 specimens). Twelve of those species were social and soil-nesting (*Bombus* spp. (Apidae), *Andrena* spp. (Andrenidae), *Halictus* spp. and *Lasioglossum (Evylaeus)* spp. (Halictidae)). Eight were solitary and soil-nesting (*Andrena bicolor* and *A. minutula* (Andrenidae), *Anthophora plumipes* and *Tetralonia malvae* (Apidae), *H. scabiosae*, *L. villosulum*, *L. nitidulum* and *L. leucozonium* (Halictidae)) and two were solitary and cavity-nesting (*Hylaeus communis* (Colletidae) and *Osmia cornuta* (Apidae)). The three most abundant species were *Lasioglossum politum* (1045 specimens; 8% of the total), *L. malachurum* (837 specimens; 6.5%), and *L. pauxillum* (566 specimens; 4.5%; Figure 2). Those three species are social, short-tongued, and soil-nesting.

### Abundance and species richness

**Figure 3:**
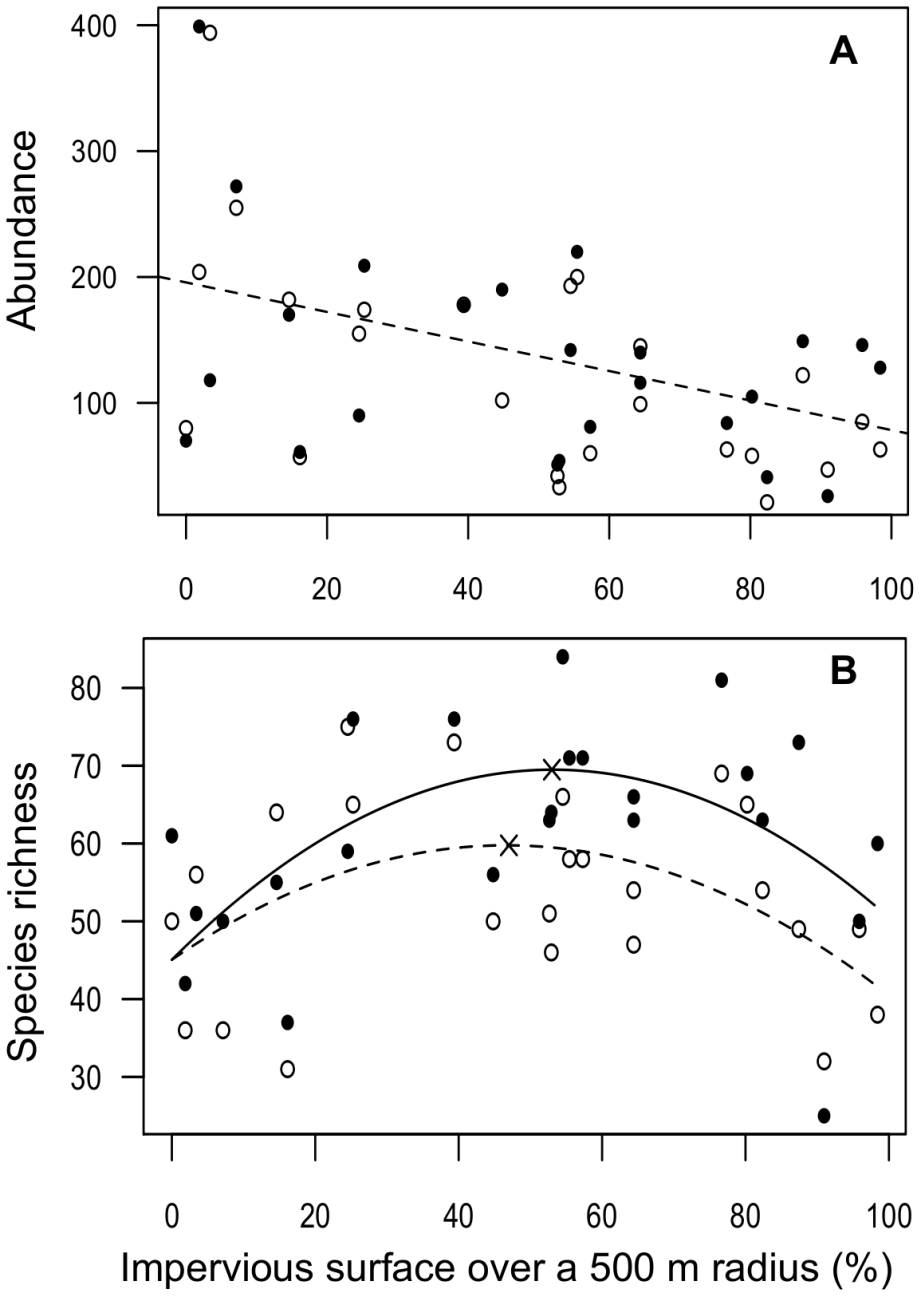
Effect of impervious surface percentage within 500 m on the abundance and species richness of bees. A. Abundance of bees (filled circles = 2011, open circles = 2012); B. Species richness of bees (filled circles and full line = 2011, open circles and dashed line = 2012). Model for species richness  =  Impervious surface (Year) + Impervious surface ^2^ (Year).

Based upon correlation analyses, among each set of significantly correlated variables, we retained only the one that gave the lowest AIC to explain abundance and species richness. In doing so, Impervious surface, Connectivity of open area, Plant diversity and Connectivity of wooded area were the sole variables that were retained in models and these three were not correlated among one another. We further introduced a quadratic term in our model (Impervious surface ^2^) to account for a non-linear pattern of the observed relationship between species richness and Impervious surface. The forward selection based on AIC enabled us to keep the variables with the greatest explanatory power in our models (Table 2). Impervious surface had a negative linear effect on abundance and a quadratic effect on species richness within the 500 m and 1000 m radii (Figure 3). Based on the quadratic models with Impervious surface only, the maximum predicted number of bee species was 69 species at a site with 53% impervious surface within 500 m in 2011 and 60 species at a site with 47% impervious surface within 500 m in 2012 (Figure 3.B). Three of the four sites with the lowest species richness over both years had low proportions of impervious surface (<12%), and high proportions of agricultural land cover (70% to 94%). Connectivity of open area had a positive effect on species richness within 1000 m (Table 2). Within 2000 m, the quadratic effect of Impervious surface on species richness was not significant, but the linear effect was, and the variable with the highest explanatory power for abundance was Connectivity of wooded area (Table 2). Plant diversity was not significant in any model.

**Table 2:** Generalized linear models for bee abundance and species richness depending on landscape variables.

Dependent variable	Radius (m)	AIC*	Independent variable	F value	*p*
Abundance	500	13.14	Impervious surface	F_2,45_ = 6.54	**0.003 (−)**
			Impervious surface^2^		
			Connectivity of open area		
			Connectivity of wooded area		
			Plant diversity		
Abundance	1000	18.31	Impervious surface	F_2,45_ = 3.57	0.036 (−)
			Impervious surface^2^		
			Connectivity of open area		
			Connectivity of wooded area		
			Plant diversity		
Abundance	2000	19.19	Impervious surface		
			Impervious surface^2^		
			Connectivity of open area		
			Connectivity of wooded area	F_2,45_ = 3.1	0.055 (+)
			Plant diversity		
Species richness	500	378.8	Impervious surface	F_2,45_ = 3.4	0.043 (+)
			Impervious surface^2^	F_2,43_ = 7.8	**0.001 (−)**
			Connectivity of open area		
			Connectivity of wooded area		
			Plant diversity		
Species richness	1000	377.23	Impervious surface	F_2,45_ = 3.5	0.039 (+)
			Impervious surface^2^	F_2,43_ = 3.36	0.045 (−)
			Connectivity of open area	F_2,41_ = 7.66	**0.002 (+)**
			Connectivity of wooded area		
			Plant diversity		
Species richness	2000	388.45	Impervious surface	F_2,45_ = 3.25	0.048 (+)
			Impervious surface^2^		
			Connectivity of open area		
			Connectivity of wooded area		
			Plant diversity		

Results of generalized linear models with abundance or species richness as dependent variables and landscape variables as independent variables. The effect of independent variables was nested in the year to account for interannual.

*AIC = Akaike Information Criterion.

P-value significant after the Bonferroni correction (i.e. *p*×3) has been applied are written in bold.

After the Bonferroni correction (*p×3*), the effect of Impervious surface on abundance was still significant within 500 m but not anymore within 1000 m. For species richness, the factors with a significant effect after the Bonferroni correction were the quadratic function of Impervious surface within 500 m and the Connectivity of open area within 1000 m. The best model fit was achieved for the 1000 m radius model (AIC = 377.23), though the low ΔAIC between the 1000 m and the 500 m models (<2, Table 2) indicates that both models are equally well supported by the data. For subsequent analyses, we kept Impervious surface and Impervious surface ^2^ as explanatory variables, and 500 m as the most relevant focus scale.

### Bee community composition and structure

**Figure 4:**
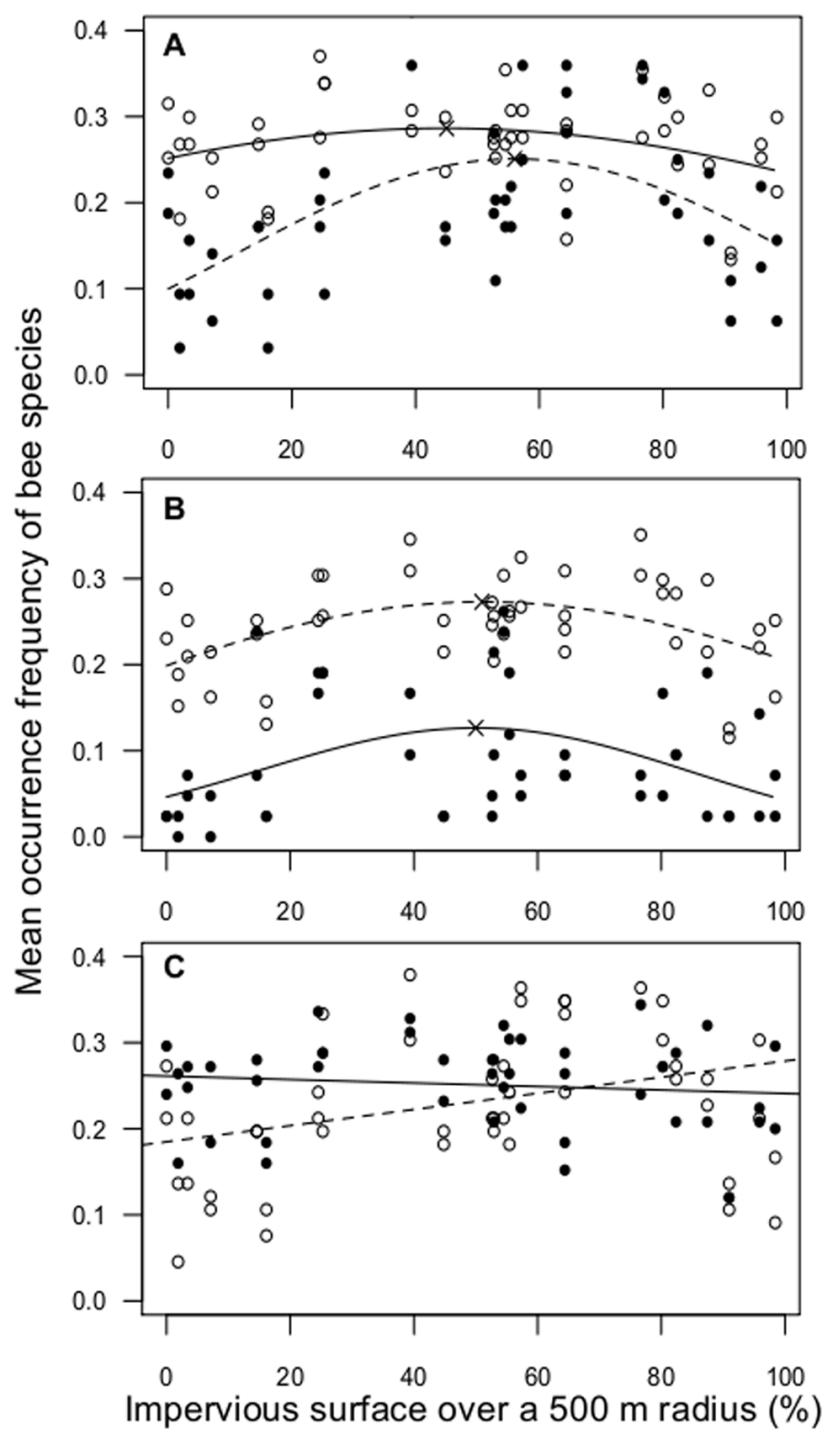
Effect of the proportion of impervious surface within 500 m on species occurrence based upon functional traits. A. Nesting behavior (filled circles and full line  =  cavity-nesting species, open circles and dashed line  =  soil-nesting species); B. Parasitic or host behavior (filled circles and full line  =  parasitic species, open circles and dashed line  =  host species) species; C. Tongue length (filled circles and full line  =  short-tongued species, open circles and dashed line  =  long-tongued species).

The occurrence frequency of bee species based on their functional traits was analyzed with selected GLM at the 500 m radius also (Table 3). The occurrence frequency of bees depending on their nesting behavior and their parasitism had a quadratic relation with Impervious surface (Figure 4.A and 4.B). The effect was higher for cavity-nesting than for soil-nesting species. The occurrence frequency of bees was highest in sites with an average of 50% impervious surface for parasitic species (Figure 4.B) and of 56% impervious surface for cavity-nesting species. The occurrence frequency of bees depending on their tongue length changed with increasing Impervious surface as there were more long-tongued species (*F_2,4463_* = 4316.7, *p*<0.001) in urbanized sites (Figure 4.C). Connectivity of open area had no effect on any functional traits. There was no effect of any landscape variable on social behavior and body size (ITD).

**Table 3:** Generalized linear models for the occurrence frequency of bee species depending on functional traits and landscape variables within 500 m.

Functionnal traits	Landscape variables	Residual deviance	*p*
Body size	Impervious surface	NS*	NS
	Impervious surface^2^	NS	NS
Nesting	Impervious surface	F_2,9159_ = 10079	**0.003 (+)**
	Impervious surface^2^	F_2,9157_ = 10063	**<0.001 (−)**
Parasitism	Impervious surface	NS	NS
	Impervious surface^2^	F_2,11173_ = 11342	**0.0076 (−)**
Sociality	Impervious surface	NS	NS
	Impervious surface^2^	NS	NS
Tongue length	Impervious surface	F_2,9159_ = 10125	**<0.001 (−)**
	Impervious surface^2^	F_2,9157_ = 10118	0.032 (+)

Results of generalized linear models with the occurrence frequency of bee species as dependent variables and landscape variables in interaction with functional traits as independent variables. The effect of independent variables was nested in the year to account for interannual. The effect of quadratic term of impervious surface proportion (Impervious surface ^2^) was higher on cavity-nesting than on soil-nesting bee species, and on non-parasitic than on parasitic bees species. The effect of impervious surface proportion was higher for long-tongued than for short-tongued species.

*NS = non significant.

P-value significant after the Bonferroni correction has been applied (i.e. *p×5*) are written in bold.

## Discussion

Our study aimed to investigate the impact of urbanization on wild bee communities. We found there were fewer individuals in sites with higher levels of urbanization, and there were more species in sites with an intermediate proportion of impervious surface. In addition, the composition of the wild bee community changed in relation to the nesting behavior of the species along the urbanization gradient.

Over two years of survey using both sweep nets and pan traps to study the effect of urbanization on the wild bee community, 291 bee species were recorded, which represents nearly 79% of the predicted number of species in the study area. Intensive sampling of bees usually leads to low number of singletons because the numbers of bee specimens and that of singletons are negatively correlated [65]. Indeed, our number of singletons represented 20% of our total number of species, which is low compared to the average of 28% (range 9-54%) recorded in 44 studies of bee communities over a range of temporal and spatial scales [65]. This suggests that the bee fauna in Grand Lyon was thoroughly surveyed or that the requirements of rare bee species (floral or nesting resources) may not be present in our study area, so these species were not detected even as singletons.

This figure of 291 accounts for nearly a third of the 912 wild bee species known in France [66]. In comparison, 262 bee species were recorded by net-collecting over 5 years in about 20 localities within the city of Berlin in Germany [9], that is 46% of the reportedly 574 wild bee species in this country [66]. In the city center and suburbs of Poznań, Poland, 104 bee species (or 19% of the national total of 537 [66]) were collected by sampling bees with yellow pan traps and insect nets every 7–10 days from April to September for 3 years (2006–2008) [23]. While direct comparison between these figures and ours is not possible due to the differences in the methodology used, it indicates nevertheless that the Lyon area did harbor a diverse bee fauna. This result may be linked to the geographical location of the Grand Lyon which is at the temperate-Mediterranean interface [67]. Climate has an important role in the establishment of wild bee communities and Mediterranean climate is known to be favorable for wild bees [68].

Parasitic bee community structure follows that of the remaining bee community, since their species richness and abundance depend on those of their hosts [69]. Indeed, several studies suggest that parasitic species are good indicators of ecosystem health and stability [70]–[75]. In our study, parasitic species represented 17% of all species. By comparison, Banaszak-Cibicka and Żmihorski (2012) found 12% parasitic species over a total of 104 species in the city of Poznań, Poland, which has 560 000 inhabitants over 261.8 km^2^ and is distant of 1469 km from Lyon [23]. The proportion of parasitic species at a national level is similar in Poland (23%, 122 species) and in France (21%, 195) (*χ^2^* = 0.27, *df* = 1, *p* = 0.6). However, the proportion of parasitic species captured in urban areas, with respect to the species proportions at the national scale, was significantly greater in our study in France compared to the Polish one (Mantel-Haenszel: *χ^2^* = 7.3, *df* = 1, *p*<0.01). The relationship between the number of parasitic bee species and the proportion of impervious surface was curvilinear with a maximum at an intermediate proportion of impervious surface (50%). Guild profiles are specific to habitats, and disturbance do not have the same effect on different guilds [55], [76], [77]. Parasitic bees play a stabilizing role in bee communities [69], [70]. They are the first to respond to disturbances. Therefore, a high diversity of parasitic species may reflect a higher stability and a higher diversity of habitats in these landscapes.

We found that an increasing proportion of impervious surface negatively affected bee abundance. Soil-nesting bees represented 86% of the total number of specimens recorded in our study and also the largest number of species. Indeed, these species represented 63% of the total species richness along our urbanization gradient, even if the occurrence frequency of soil-nesting bees slowly decreased with increasing proportion of impervious surface. In urban sites, resources for ground-nesting bees are less abundant because of the predominance of impervious surface and this would likely jeopardize the establishment of soil-nesting bees. Furthermore, 15 of the 25 most abundant species were soil-nesting and social, so that these species may be over-represented in our pan trap captures simply owing to their social behavior. Indeed, social bee species tend to be active for a longer period than solitary species. The attractiveness pattern of pan traps may also explain this negative relationship between bee abundance and urbanization. The effectiveness of pan traps is inversely related to the abundance of flowers in their surroundings [35], [78]. In urban green areas where we exposed our pan traps, flowers were concentrated in flowerbeds that usually provide a large and year-long floral display to bees [15]. But in sites with less impervious surface, bees probably had to fly longer distances between adjacent forage resources and pan traps attractiveness may therefore have been better in these habitats.

To predict diversity and species composition changes in urban systems, urban areas can be modeled using the disturbance heterogeneity model ([79]) [80]. This model specifically incorporates spatial (as opposed to temporal) disturbances to account for increased habitat diversity and suggests that when the proportion of disturbed habitat reaches 50%, the area has maximal heterogeneity [80]. When the proportion of disturbed habitat increases or decreases beyond this value, the area becomes more homogeneous. Following this disturbance heterogeneity model, maximum heterogeneity should lead to peak species diversity at 50% impervious surface [80], since such surfaces can be considered as disturbed and mainly unusable habitats for bees, especially ground-nesting ones. Indeed, urban disturbances eliminate potential ground nesting habitats because of impervious surface [80]. In our study, the response of bee diversity to urbanization was consistent with this model with maximum species diversity at 53% impervious surface in 2011 and 47% in 2012. The city center is largely composed of abiotic elements such as paved streets, sidewalks, and buildings with planted trees and flowerbeds usually as sole green elements. In contrast, the periurban landscape, although heavily disturbed too, usually includes many gardens and green recreation areas, as well as roadsides with vegetation that provide more suitable habitats for ground-nesting bees. Fully urbanized areas may thus provide fewer resources for bees in comparison with periurban areas that have around 50% impervious surface and, thus, can harbor more diverse floral and nesting resources [15].

In our study, we took botanical information into account by recording plant species richness over two 50 m perpendicular transects at each site. This variable, which included all flowering plant species (Spermaphytes), had no effect on bee species richness, which was surprising given the importance of floral diversity on bee diversity [96]. We probably should have focused on the diversity of flowers that are actually visited by bees to better assess the importance of this factor.

In addition to richness and abundance, we studied the changes of the community structure along the urbanization gradient by the studying functional traits of bee species. Within all families, bees present a diverse assemblage of functional traits [19], [20], which makes it difficult to characterize the community as a whole, especially when habitat comparisons are the topic of investigation [69]. Urbanized landscapes usually include some green areas that can provide forage resources for a diversity of wild bees [11], [81]. These landscapes may also contain diverse nesting opportunities, such as bare soil, dead stems and manmade cavities [12], [24]. In our study, soil-nesting and short-tongued bees were little affected by urbanization, whereas cavity-nesting species and long-tongued species were more numerous in moderately and highly urbanized areas, respectively. For nesting behavior, our result is in agreement with several studies that report a greater abundance of cavity-nesting bee species in periurban and urban areas compared to sites with less impervious surface [3], [7], [13]. Even if cavity-nesting species richness reached a maximum in sites with intermediate proportion of impervious surface, there were more cavity-nesting bee species in urbanized areas than in more natural ones. The hypothesis here is that cavity-nesting bees may find more nesting resources in urbanized habitats because of manmade cavities [12], [82]. Concerning tongue-length, long-tongued species can visit flowers with short or long corolla [83], so they may be less affected than short-tongued species by the changes in floral resources that may occur over an urbanization gradient. Overall, these patterns were not unexpected, since nesting behavior and tongue length are not independent functional traits. Indeed, most ground nesting species were Halictidae and Andrenidae, which are also short-tongued, while cavity nesting species were Megachilidae, which are mostly long-tongued.

Flight distance is related to body size [43], [44], [84], and it influences the ability of bees to recolonize disturbed sites [19]. Thus, we expected larger species to be less affected by urbanization or by the connectivity of open area [85]. Yet, none of the landscape variables had a significant effect on the body size of bees along our urbanization gradient. Although, this functional trait is important for determining species responses to landscape changes, there are opposing predictions for these responses [3], [85]–[87]. Even if small species (<3 mm; [85]) have limited abilities to recolonize disturbed habitats, this may be counterbalanced by the fact that they require less food resources than large species (>5 mm; [85]) and so may be better able to maintain their populations in disturbed habitats, such as urbanized areas [19]. It is known that social bees have a better adaptability to disturbance than solitary species [23], and that solitary species are more sensitive to disturbance in temperate grasslands [88]. However, none of the landscape variables had a significant effect on the proportion of social species. In our study, most of the social bees were soil-nesting (94%), and we found that cavity-nesting species were more numerous in urbanized sites, thus this soil-nesting preference may counterbalance the social status.

Among many human activities that promote biotic homogenization, urbanization is one of the strongest [1]. Urban biotic communities reflect adaptations to the physical environment as well as the biotic interactions (such as predation and competition) that occur in these environments [89], [90]. Species along an urban gradient can be classified into three distinct categories reflecting their response to urbanization [91], [92]: avoidance, adaptation, and exploitation [93]. Witte *et al.* (1985) even use the terms ‘urbanophobes’ and ‘urbanophiles’ to describe negative and positive responses to urbanization, respectively, and Kuhn *et al.* (2004) added the term ‘moderately urbanophilic’ species that are most abundant in sites with intermediate proportion of impervious surface [92], [94]. Following this terminology, parasitic species and cavity nesting could be qualified as ‘moderately urbanophilic’, and long-tongued species as ‘urbanophiles’.

Urbanization and agricultural intensification are two human activities that result in extensive changes of the landscape and its environment, and lead to the destruction or the fragmentation of natural habitats [24]. In our study, three of the four sites with the lowest species richness had a high proportion of agricultural land cover (range 70–94%). Our urbanized sites thus seemed more favorable to a diverse wild bee fauna than agricultural ones. High spatial and temporal instability of agricultural sites, associated with intensive agricultural practices (e.g. soil plowing, pesticide use, crop rotation, landscape simplification) are the main causes of bee diversity loss in farmland areas [95], [96]. Further studies are needed to test the hypothesis that, in a given context of fragmentation, urbanized landscapes are more favorable to a species-rich wild bee community than agricultural ones.

Overall, our results suggest that urbanized sites can provide forage and nesting resources for a large community of wild bee species, even if the landscapes with an intermediate proportion of impervious surface have a more diverse and abundant bee fauna. Flagship species are defined as ‘known charismatic species that serve as a symbol or focus point to raise environmental consciousness’ [97]. Although their individual species may be difficult to identify [98], bees can collectively be considered as a flagship group of species and used to raise the awareness of city-dwellers to biodiversity, as we observed in this study (http://www.urbanbees.eu). Indeed, the loss of a charismatic species can affect people more than the loss of habitat, even when the loss of habitat is the very threat to the species [99]. Also, because bees are a key group of pollinators worldwide for both wild and cultivated entomophilous plants [100], [101], bees can be readily used to illustrate the importance of ecosystem services, ecosystem functions and natural capital. Focusing public attention on city-dwelling species such as wild bees provides great opportunities to demonstrate the importance of conservation to society. The perception of wildlife by society is crucial for effective conservation of biodiversity [102], [103], and, since today 74% of the Europe's population lives in cities [104], it is both essential and urgent to raise the awareness of urban citizens on the importance for biodiversity conservation [105].

## Supporting Information

Figure S1 **Results of the principal component analyses on the landscapes variables over a 500 m radius.**
(PDF)

Table S1 **Information on the 24 sites of the study.**
(PDF)

Table S2 **List of recorded bee species list and their functional traits.**
(PDF)

Table S3 **Significant correlation between landscape variables.**
(PDF)
