# Supplementary figures and images for: Decreasing Abundance, Increasing Diversity and Changing Structure of the Wild Bee Community (Hymenoptera: Anthophila) along an Urbanization Gradient
Source: PLoS One. 2014 Aug 13;9(8):e104679. doi: 10.1371/journal.pone.0104679 (PMC4131891; doi:10.1371/journal.pone.0104679)

Dim 2 (18.96%)

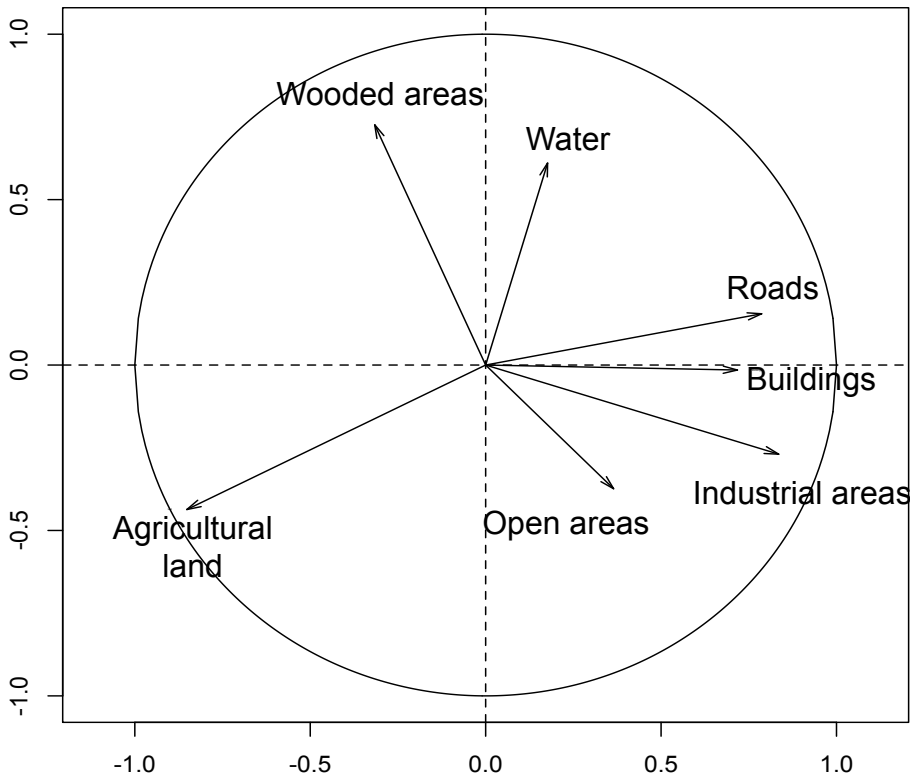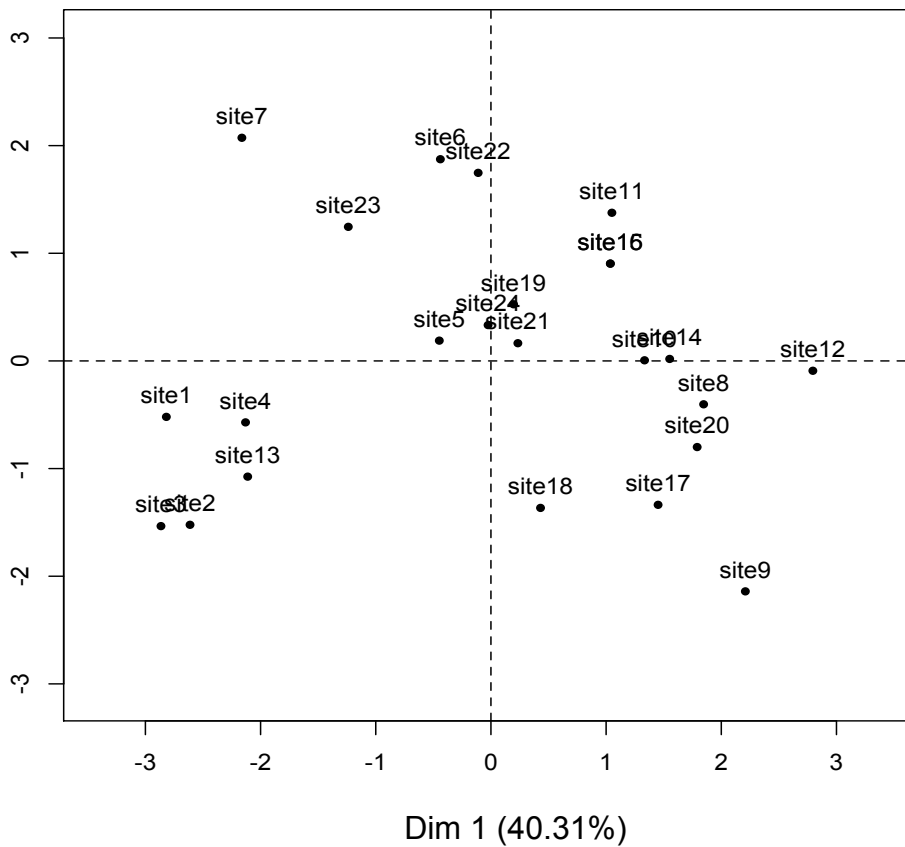

Supplement: Figure S1 — Results of the principal component analyses on the landscapes variables over a 500 m radius. (PDF) [file pone.0104679.s001.pdf]
