# Supplementary material for: Decreasing Abundance, Increasing Diversity and Changing Structure of the Wild Bee Community (Hymenoptera: Anthophila) along an Urbanization Gradient
Source: PLoS One. 2014 Aug 13;9(8):e104679. doi: 10.1371/journal.pone.0104679 (PMC4131891; doi:10.1371/journal.pone.0104679)
Supplement: Table S1 — Information on the 24 sites of the study. (PDF) [file pone.0104679.s002.pdf]

Table S1: Information on the 24 sites of the study.

Site	City	Latitude	Longitude	Altitude (m)	UTM/WGS84 ellipsoid	Proportion of impervious surface (%)	Number of species	Number of specimens in pan-traps	Authority which provided the permit to sample bees
1	TALUYERS	45°37'09.4"N	4°43'50.1"E	187	31TFL 34919 53205	0.88	87	151	CREN Rhône-Alpes[1]
2	GENAS	45°44'16.6"N	5°3'13.4"E	225	31TFL 59773 66983	4.4	69	527	Farmer
3	FEYZIN	45°40'30"N	4°52'57.3"E	214	31TFL 46623 59662	8.14	58	604	Farmer
4	ST GERMAIN AU MT D'OR	45°53'10.30"N	4°47'12.60"E	199	31TFL 47461 81606	15.56	54	118	Farmer
5	BALAN	45°49'23.07"N	5°7'23.12"E	189	31TFL 64918 76582	26.38	72	292	CREN Rhône-Alpes[1]
6	CREPIEUX LA PAPE	45°48'06.1"N	4°53'51"E	172	31TFL 47451 73766	34.38	98	383	Veolia[2] and CREN Rhône-Alpes[1]
7	MEYZIEU	45°48'03.1"N	5°00'03.4"E	179	31TFL 55492 73869	12.23	80	513	Syndicat Mixte pour l'Aménagement et la Gestion du Grand Parc de Miribel Jonage[3], Société Anonyme d'Economie Mixte pour la gestion et l'Animation des équipements de Plein air et de Loisirs du Parc de Miribel Jonage[4], and EARL de l'Abbaye
8	LYON	45°44'52.45"N	4°53'42.3"E	165	31TFL 47405 67785	88.82	88	271	City of Lyon
9	SAINT-PRIEST	45°41'46.9"N	4°56'35.2"E	219	31TFL 51280 62148	85.52	99	163	City of Saint Priest
10	VILLEURBANNE	45°47'17.4"N	4°52'55.6"E	172	31TFL 46291 72234	48.04	96	420	City of Villeurbanne
11	LYON	45°47'3.9"N	4°51'22.1"E	188	31TFL 44282 71770	54.14	99	335	City of Lyon
12	LYON	45°46'20.92"N	4°49'47.03"E	213	31TFL 42259 70397	79.73	42	74	City of Lyon
13	CAILLOUX SUR FONTAINE	45°52'20"N	4°54'00.1"E	328	31TFL 29206 69886	23.57	87	352	Farmer
14	LYON	45°46'13.27"N	4°47'45.05"E	172	31TFL 39630 70101	76.46	99	147	City of Lyon
15	VILLEURBANNE	45°46'16.35"N	4°52'47.03"E	181	31TFL 46150 70346	96.24	85	262	City of Villeurbanne
16	LYON	45°43'27.1"N	4°49'37.3"E	166	31TFL 42172 65028	75.6	85	239	City of Lyon
17	VILLEURBANNE	45°45'23.90"N	4°55'8.16"E	190	31TFL 49237 68800	98.14	68	192	City of Villeurbanne
18	FRANCHEVILLE	45°44'28.48"N	4°44'10.25"E	217	31TFL 35061 66764	86.31	87	62	Grand Lyon[5]
19	LIMONEST	45°50'02.2"N	4°46'28.8"E	395	31TFL 37826 77129	38.94	83	87	City of Limonest and Syndicat Mixte des Monts d'Or[6]
20	MEYZIEU	45°46'2.05"N	5°00'30.4"E	210	31TFL 56169 70148	94.58	74	231	City of Meyzieu
21	STE FOY LES LYON	45°44'15.66"N	4°47'9.99"E	255	31TFL 38954 66454	70.79	87	141	City of Sainte Foy-lès-Lyon
22	GRIGNY	45°36'49.29"N	4°48'1.90"E	129	31TFL 40386 52704	37.85	104	355	City of Grigny and Syndicat Mixte du Rhône, des Iles et des Lônes[7]
23	COLLONGES AU MT D'OR	45°49'49.67"N	4°49'55.19"E	249	31TFL 42288 76843	42.49	94	245	City of Collonges au Mont d'Or and Syndicat Mixte des Monts d'Or[6]
24	MARCY L'ETOILE	45°47'46.12"N	4°42'53.84"E	283	31TFL 33279 72828	31.95	77	93	VetAgro Sup[8]

[1] Conservatoire d'Espaces Naturels Rhône-Alpes (http://www.cren-rhonealpes.fr/)

[2] http://www.veoliaeau.com/

[3] http://www.grand-parc.fr/institutionnel/acteurs-symalim.asp

[4] http://www.grand-parc.fr/institutionnel/acteurs-segapal.asp

[5] http://www.grandlyon.com/

[6] http://www.montsdor.com/

[7] http://www.smiril.fr/

[8] http://www.vetagro-sup.fr/
