# Supplementary material for: Decreasing Abundance, Increasing Diversity and Changing Structure of the Wild Bee Community (Hymenoptera: Anthophila) along an Urbanization Gradient
Source: PLoS One. 2014 Aug 13;9(8):e104679. doi: 10.1371/journal.pone.0104679 (PMC4131891; doi:10.1371/journal.pone.0104679)
Supplement: Table S2 — List of recorded bee species list and their functional traits. (PDF) [file pone.0104679.s003.pdf]

Table S2: List of recorded bee species list and their functional traits.

Taxa	Family	Number of specimens	Inter-Tegular Distance (mm)	Tongue lenght	Parasitsm	Social status	Nesting
Amegilla albigena	Apidae	14	3.02	Long	Non-parasitic	Solitary	Soil
Amegilla garrula	Apidae	2	4.28	Long	Non-parasitic	Solitary	Soil
Amegilla quadrifasciata	Apidae	8	3.80	Long	Non-parasitic	Solitary	Soil
Amegilla salviae	Apidae	5	5.91	Long	Non-parasitic	Solitary	Soil
Andrena aeneiventris	Andrenidae	1	0.86	Short	Non-parasitic	NA	Soil
Andrena agilissima	Andrenidae	4	2.94	Short	Non-parasitic	Solitary	Soil
Andrena alfkenella	Andrenidae	3	1.33	Short	Non-parasitic	Solitary	Soil
Andrena alutacea	Andrenidae	1	NA	Short	Non-parasitic	Solitary	Soil
Andrena angustior	Andrenidae	4	1.88	Short	Non-parasitic	Solitary	Soil
Andrena barbilabris	Andrenidae	2	2.34	Short	Non-parasitic	Solitary	Soil
Andrena bicolor	Andrenidae	179	2.10	Short	Non-parasitic	Solitary	Soil
Andrena bimaculata	Andrenidae	5	2.72	Short	Non-parasitic	Solitary	Soil
Andrena bucephala	Andrenidae	1	2.15	Short	Non-parasitic	Social	Soil
Andrena carantonica	Andrenidae	8	2.72	Short	Non-parasitic	Social	Soil
Andrena chrysosceles	Andrenidae	21	1.97	Short	Non-parasitic	Solitary	Soil
Andrena cineraria	Andrenidae	423	2.76	Short	Non-parasitic	Social	Soil
Andrena combinata	Andrenidae	2	2.14	Short	Non-parasitic	Social	Soil
Andrena curvungula	Andrenidae	28	2.38	Short	Non-parasitic	Social	Soil
Andrena decipiens	Andrenidae	1	1.61	Short	Non-parasitic	Social	Soil
Andrena distinguenda	Andrenidae	12	1.68	Short	Non-parasitic	Solitary	Soil
Andrena dorsata	Andrenidae	111	2.03	Short	Non-parasitic	Solitary	Soil
Andrena fabrella	Andrenidae	3	0.63	Short	Non-parasitic	NA	Soil
Andrena falsifica	Andrenidae	18	1.38	Short	Non-parasitic	Solitary	Soil
Andrena flavipes	Andrenidae	351	2.36	Short	Non-parasitic	Social	Soil
Andrena florea	Andrenidae	24	2.43	Short	Non-parasitic	Social	Soil
Andrena floricola	Andrenidae	3	1.50	Short	Non-parasitic	Solitary	Soil
Andrena fulva	Andrenidae	40	2.78	Short	Non-parasitic	Social	Soil
Andrena fulvago	Andrenidae	38	2.06	Short	Non-parasitic	Social	Soil
Andrena fulvata	Andrenidae	27	1.97	Short	Non-parasitic	Solitary	Soil
Andrena granulosa	Andrenidae	1	2.11	Short	Non-parasitic	Solitary	Soil
Andrena gravida	Andrenidae	63	2.59	Short	Non-parasitic	Social	Soil
Andrena haemorrhoa	Andrenidae	108	2.63	Short	Non-parasitic	Social	Soil
Andrena hattorfiana	Andrenidae	2	2.71	Short	Non-parasitic	Solitary	Soil
Andrena helvola	Andrenidae	1	2.09	Short	Non-parasitic	Solitary	Soil
Andrena humilis	Andrenidae	6	2.23	Short	Non-parasitic	Social	Soil
Andrena labialis	Andrenidae	2	2.70	Short	Non-parasitic	Social	Soil
Andrena labiata	Andrenidae	10	1.78	Short	Non-parasitic	Social	Soil
Andrena lagopus	Andrenidae	78	2.34	Short	Non-parasitic	Solitary	Soil
Andrena lathyri	Andrenidae	5	2.68	Short	Non-parasitic	Solitary	Soil
Andrena marginata	Andrenidae	1	1.95	Short	Non-parasitic	Social	Soil
Andrena minutula	Andrenidae	154	1.43	Short	Non-parasitic	Solitary	Soil
Andrena minutuloides	Andrenidae	85	1.39	Short	Non-parasitic	Solitary	Soil
Andrena mitis	Andrenidae	2	2.31	Short	Non-parasitic	Solitary	Soil
Andrena nana	Andrenidae	1	1.58	Short	Non-parasitic	Solitary	Soil
Andrena nigroaenea	Andrenidae	23	2.84	Short	Non-parasitic	Solitary	Soil
Andrena nitida	Andrenidae	20	2.97	Short	Non-parasitic	Solitary	Soil
Andrena nitidiuscula	Andrenidae	5	1.58	Short	Non-parasitic	Solitary	Soil
Andrena nitidula	Andrenidae	1	NA	Short	Non-parasitic	NA	Soil
Andrena niveata	Andrenidae	1	1.51	Short	Non-parasitic	Solitary	Soil
Andrena nycthemera	Andrenidae	3	2.90	Short	Non-parasitic	Social	Soil
Andrena ovatula	Andrenidae	65	2.24	Short	Non-parasitic	Social	Soil
Andrena pandellei	Andrenidae	8	2.17	Short	Non-parasitic	Social	Soil
Andrena paucisquama	Andrenidae	1	2.13	Short	Non-parasitic	NA	Soil
Andrena propinqua	Andrenidae	41	2.04	Short	Non-parasitic	Solitary	Soil
Andrena pusilla	Andrenidae	68	1.39	Short	Non-parasitic	NA	Soil
Andrena ranunculi	Andrenidae	3	1.75	Short	Non-parasitic	NA	Soil
Andrena rhenana	Andrenidae	35	2.17	Short	Non-parasitic	Solitary	Soil
Andrena rufula	Andrenidae	1	1.13	Short	Non-parasitic	NA	Soil
Andrena saxonica	Andrenidae	2	0.63	Short	Non-parasitic	NA	Soil
Andrena schencki	Andrenidae	3	2.40	Short	Non-parasitic	Social	Soil
Andrena similis	Andrenidae	3	2.34	Short	Non-parasitic	Solitary	Soil
Andrena simontornyella	Andrenidae	80	1.37	Short	Non-parasitic	NA	Soil
Andrena spreta	Andrenidae	2	NA	Short	Non-parasitic	NA	Soil
Andrena strohmella	Andrenidae	16	1.58	Short	Non-parasitic	Solitary	Soil
Andrena subopaca	Andrenidae	3	1.49	Short	Non-parasitic	NA	Soil
Andrena tibialis	Andrenidae	1	2.85	Short	Non-parasitic	Solitary	Soil
Andrena trimmerana	Andrenidae	23	2.25	Short	Non-parasitic	NA	Soil
Andrena vaga	Andrenidae	16	2.76	Short	Non-parasitic	Social	Soil
Andrena ventralis	Andrenidae	38	1.60	Short	Non-parasitic	Social	Soil

Taxa	Family	Number of specimens	Inter-Tegular Distance (mm)	Tongue lenght	Parasitsm	Social status	Nesting
Andrena viridescens	Andrenidae	10	1.42	Short	Non-parasitic	Solitary	Soil
Andrena vulpecula	Andrenidae	1	1.98	Short	Non-parasitic	NA	Soil
Andrena wilkella	Andrenidae	7	2.15	Short	Non-parasitic	Solitary	Soil
Anthidiellum strigatum	Megachilidae	1	2.44	Long	Non-parasitic	Solitary	Cavity
Anthidium florentinum	Megachilidae	95	3.65	Long	Non-parasitic	NA	NA
Anthidium lituratum	Megachilidae	2	1.67	Long	Non-parasitic	Solitary	Cavity
Anthidium manicatum	Megachilidae	34	3.59	Long	Non-parasitic	Solitary	Cavity
Anthidium nanum	Megachilidae	1	NA	Long	Non-parasitic	NA	NA
Anthidium oblongatum	Megachilidae	26	2.67	Long	Non-parasitic	Solitary	Cavity
Anthophora bimaculata	Apidae	20	5.64	Long	Non-parasitic	Solitary	Soil
Anthophora furcata	Apidae	2	NA	Long	Non-parasitic	Solitary	Cavity
Anthophora plumipes	Apidae	282	4.33	Long	Non-parasitic	Solitary	Soil
Bombus campestris	Apidae	4	2.20	Long	Parasitic	-	-
Bombus hortorum	Apidae	41	5.77	Long	Non-parasitic	Social	Soil
Bombus humilis	Apidae	22	4.77	Long	Non-parasitic	Social	Soil
Bombus hypnorum	Apidae	18	4.71	Long	Non-parasitic	Social	Cavity
Bombus lapidarius	Apidae	325	4.97	Long	Non-parasitic	Social	Soil
Bombus lucorum	Apidae	21	4.49	Long	Non-parasitic	Social	Soil
Bombus magnus	Apidae	1	2.30	Long	Non-parasitic	NA	NA
Bombus pascuorum	Apidae	490	4.32	Long	Non-parasitic	Social	Soil
Bombus pratorum	Apidae	43	4.61	Long	Non-parasitic	Social	Soil
Bombus ruderatus	Apidae	10	4.00	Long	Non-parasitic	Social	Soil
Bombus sylvarum	Apidae	9	4.44	Long	Non-parasitic	Social	Cavity
Bombus sylvestris	Apidae	11	4.27	Long	Parasitic	-	-
Bombus terrestris	Apidae	341	6.15	Long	Non-parasitic	Social	Soil
Bombus vestalis	Apidae	16	5.68	Long	Parasitic	-	-
Ceratina callosa	Apidae	2	1.02	Long	Non-parasitic	Solitary	Soil
Ceratina cucurbitina	Apidae	91	1.45	Long	Non-parasitic	Solitary	Cavity
Ceratina cyanea	Apidae	41	1.39	Long	Non-parasitic	Solitary	Cavity
Ceylalictus variegatus	Halictidae	1	0.67	Short	Non-parasitic	NA	Soil
Chelostoma florisomne	Megachilidae	40	1.79	Long	Non-parasitic	Solitary	Cavity
Chelostoma proximum	Megachilidae	2	0.60	Long	Non-parasitic	NA	NA
Chelostoma rapunculi	Megachilidae	13	1.63	Long	Non-parasitic	Solitary	Cavity
Coelioxys echinata	Megachilidae	1	NA	Long	Parasitic	-	-
Coelioxys elongata	Megachilidae	2	NA	Long	Parasitic	-	-
Coelioxys inermis	Megachilidae	1	NA	Long	Parasitic	-	-
Coelioxys quadridentata	Megachilidae	2	1.13	Long	Parasitic	-	-
Colletes cunicularius	Colletidae	22	3.57	Short	Non-parasitic	Solitary	Soil
Colletes daviesanus	Colletidae	18	2.46	Short	Non-parasitic	Solitary	Soil
Colletes fodiens	Colletidae	2	2.60	Short	Non-parasitic	Solitary	Soil
Colletes gallicus	Colletidae	2	2.88	Short	Non-parasitic	Solitary	Soil
Colletes hederae	Colletidae	23	3.23	Short	Non-parasitic	Solitary	Soil
Colletes similis	Colletidae	9	2.37	Short	Non-parasitic	Solitary	Soil
Dasypoda hirtipes	Melittidae	46	5.70	Short	Non-parasitic	Solitary	Soil
Epeolus cruciger	Apidae	1	1.86	Long	Parasitic	-	-
Epeolus schummeli	Apidae	1	NA	Long	Non-parasitic	Solitary	Soil
Epeolus variegatus	Apidae	11	1.44	Long	Parasitic	-	-
Eucera interrupta	Apidae	4	0.67	Long	Non-parasitic	Solitary	Soil
Eucera longicornis	Apidae	4	3.48	Long	Non-parasitic	Solitary	Soil
Eucera nigrescens	Apidae	29	3.52	Long	Non-parasitic	Solitary	Cavity
Halictus eurygnathus	Halictidae	2	NA	Short	Non-parasitic	Solitary	Soil
Halictus gavarnicus	Halictidae	3	1.15	Short	Non-parasitic	NA	Soil
Halictus langobardicus	Halictidae	9	4.24	Short	Non-parasitic	NA	Soil
Halictus maculatus	Halictidae	83	1.55	Short	Non-parasitic	Social	Soil
Halictus Monilapis gr.	Halictidae	212	2.04	Short	Non-parasitic	NA	NA
Halictus quadricinctus	Halictidae	108	2.93	Short	Non-parasitic	Solitary	Soil
Halictus rubicundus	Halictidae	9	2.18	Short	Non-parasitic	Social	Soil
Halictus scabiosae	Halictidae	267	2.43	Short	Non-parasitic	Solitary	Soil
Halictus simplex	Halictidae	21	1.90	Short	Non-parasitic	Solitary	Soil
Halictus smaragdulus	Halictidae	14	1.03	Short	Non-parasitic	Social	Soil
Halictus subauratus	Halictidae	236	1.62	Short	Non-parasitic	Social	Soil
Halictus tumulorum	Halictidae	22	1.49	Short	Non-parasitic	Social	Soil
Heriades crenulatus	Megachilidae	16	1.53	Long	Non-parasitic	Solitary	Cavity
Heriades truncorum	Megachilidae	67	1.51	Long	Non-parasitic	Solitary	Cavity
Hoplitis adunca	Megachilidae	61	2.67	Long	Non-parasitic	Solitary	Cavity
Hoplitis lepeletieri	Megachilidae	1	NA	Long	Non-parasitic	Solitary	Cavity
Hoplitis leucomelana	Megachilidae	3	0.64	Long	Non-parasitic	Solitary	Cavity
Hoplitis ravouxi	Megachilidae	1	NA	Long	Non-parasitic	Solitary	Cavity
Hoplitis tridentata	Megachilidae	1	NA	Long	Non-parasitic	Solitary	Cavity
Hylaeus angustatus	Colletidae	1	1.06	Short	Non-parasitic	Solitary	Cavity
Hylaeus annularis	Colletidae	1	1.67	Short	Non-parasitic	Solitary	Cavity

Taxa	Family	Number of specimens	Inter-Tegular Distance (mm)	Tongue lenght	Parasitsm	Social status	Nesting
Hylaeus brevicornis	Colletidae	11	1.02	Short	Non-parasitic	Solitary	Cavity
Hylaeus clypearis	Colletidae	15	0.86	Short	Non-parasitic	Solitary	Cavity
Hylaeus communis	Colletidae	157	1.23	Short	Non-parasitic	Solitary	Cavity
Hylaeus cornutus	Colletidae	2	1.42	Short	Non-parasitic	Solitary	Cavity
Hylaeus difformis	Colletidae	1	1.50	Short	Non-parasitic	Solitary	Cavity
Hylaeus dilatatus	Colletidae	6	1.34	Short	Non-parasitic	Solitary	NA
Hylaeus duckei	Colletidae	8	2.53	Short	Non-parasitic	Solitary	Cavity
Hylaeus gibbus	Colletidae	35	1.43	Short	Non-parasitic	Solitary	Cavity
Hylaeus gredleri	Colletidae	42	1.23	Short	Non-parasitic	Solitary	Cavity
Hylaeus hyalinatus	Colletidae	62	1.39	Short	Non-parasitic	Solitary	Cavity
Hylaeus incongruus	Colletidae	33	2.69	Short	Non-parasitic	Solitary	NA
Hylaeus leptocephalus	Colletidae	25	2.28	Short	Non-parasitic	Solitary	Cavity
Hylaeus nigritus	Colletidae	1	1.62	Short	Non-parasitic	Solitary	Cavity
Hylaeus paulus	Colletidae	2	NA	Short	Non-parasitic	Solitary	Cavity
Hylaeus pictipes	Colletidae	54	1.05	Short	Non-parasitic	Solitary	Cavity
Hylaeus punctatus	Colletidae	47	1.19	Short	Non-parasitic	Solitary	Cavity
Hylaeus punctulatissimus	Colletidae	2	1.47	Short	Non-parasitic	Solitary	Cavity
Hylaeus signatus	Colletidae	1	1.81	Short	Non-parasitic	Solitary	Cavity
Hylaeus sinuatus	Colletidae	7	1.32	Short	Non-parasitic	Solitary	Cavity
Hylaeus tyrolensis	Colletidae	6	NA	Short	Non-parasitic	NA	NA
Hylaeus variegatus	Colletidae	7	2.88	Short	Non-parasitic	Solitary	Cavity
Lasioglossum aeratum	Halictidae	1	1.06	Short	Non-parasitic	Social	Soil
Lasioglossum albipes	Halictidae	3	1.64	Short	Non-parasitic	Social	Soil
Lasioglossum albocinctum	Halictidae	35	2.52	Short	Non-parasitic	Social	Soil
Lasioglossum bluethgeni	Halictidae	20	1.43	Short	Non-parasitic	NA	Soil
Lasioglossum brevicorne	Halictidae	1	1.33	Short	Non-parasitic	NA	Soil
Lasioglossum calceatum	Halictidae	167	1.82	Short	Non-parasitic	Social	Soil
Lasioglossum discum	Halictidae	1	4.40	Short	Non-parasitic	NA	Soil
Lasioglossum euboeense	Halictidae	34	4.30	Short	Non-parasitic	NA	Soil
Lasioglossum fulvicorne	Halictidae	74	1.31	Short	Non-parasitic	Solitary	Soil
Lasioglossum glabriusculum	Halictidae	29	0.73	Short	Non-parasitic	Social	Soil
Lasioglossum griseolum	Halictidae	46	0.93	Short	Non-parasitic	NA	Soil
Lasioglossum interruptum	Halictidae	61	2.23	Short	Non-parasitic	Social	Soil
Lasioglossum laevigatum	Halictidae	1	1.84	Short	Non-parasitic	Solitary	Soil
Lasioglossum laticeps	Halictidae	284	1.48	Short	Non-parasitic	Social	Soil
Lasioglossum lativentre	Halictidae	21	1.55	Short	Non-parasitic	Solitary	Soil
Lasioglossum leucozonium	Halictidae	138	1.91	Short	Non-parasitic	Solitary	Soil
Lasioglossum limbellum	Halictidae	6	1.32	Short	Non-parasitic	Solitary	Soil
Lasioglossum lineare	Halictidae	395	1.42	Short	Non-parasitic	Social	Soil
Lasioglossum lucidulum	Halictidae	6	1.61	Short	Non-parasitic	Solitary	Soil
Lasioglossum majus	Halictidae	17	3.88	Short	Non-parasitic	Solitary	Soil
Lasioglossum malachurum	Halictidae	838	1.79	Short	Non-parasitic	Social	Soil
Lasioglossum marginatum	Halictidae	472	1.59	Short	Non-parasitic	Social	Soil
Lasioglossum mediterraneum	Halictidae	3	3.34	Short	Non-parasitic	NA	Soil
Lasioglossum mesosclerum	Halictidae	4	1.31	Short	Non-parasitic	NA	Soil
Lasioglossum minutissimum	Halictidae	21	0.86	Short	Non-parasitic	Solitary	Soil
Lasioglossum minutulum	Halictidae	1	1.25	Short	Non-parasitic	Solitary	Soil
Lasioglossum morio	Halictidae	358	1.10	Short	Non-parasitic	Social	Soil
Lasioglossum nigripes	Halictidae	6	1.97	Short	Non-parasitic	Social	Soil
Lasioglossum nitidulum	Halictidae	149	1.24	Short	Non-parasitic	Solitary	Soil
Lasioglossum pallens	Halictidae	12	1.59	Short	Non-parasitic	Solitary	Soil
Lasioglossum pauperatum	Halictidae	7	1.20	Short	Non-parasitic	NA	Soil
Lasioglossum pauxillum	Halictidae	565	1.24	Short	Non-parasitic	Social	Soil
Lasioglossum politum	Halictidae	1045	0.82	Short	Non-parasitic	Social	Soil
Lasioglossum prasinum	Halictidae	3	1.72	Short	Non-parasitic	Solitary	Soil
Lasioglossum punctatissimum	Halictidae	81	1.23	Short	Non-parasitic	NA	Soil
Lasioglossum puncticolle	Halictidae	1	1.49	Short	Non-parasitic	Social	Soil
Lasioglossum pygmaeum	Halictidae	32	1.30	Short	Non-parasitic	NA	Soil
Lasioglossum sabulosum	Halictidae	3	1.00	Short	Non-parasitic	Solitary	Soil
Lasioglossum semilucens	Halictidae	2	0.36	Short	Non-parasitic	NA	Soil
Lasioglossum sexnotatum	Halictidae	1	2.07	Short	Non-parasitic	Solitary	Soil
Lasioglossum sexstrigatum	Halictidae	2	2.55	Short	Non-parasitic	Solitary	Soil
Lasioglossum subhirtum	Halictidae	295	1.32	Short	Non-parasitic	NA	Soil
Lasioglossum transitorium	Halictidae	1	1.27	Short	Non-parasitic	NA	Soil
Lasioglossum tricinctum	Halictidae	4	1.25	Short	Non-parasitic	Solitary	Soil
Lasioglossum villosulum	Halictidae	168	1.33	Short	Non-parasitic	Solitary	Soil
Lasioglossum xanthopus	Halictidae	5	2.37	Short	Non-parasitic	Solitary	Soil
Lasioglossum zonulum	Halictidae	24	1.91	Short	Non-parasitic	Solitary	Soil
Lithurgus chrysurus	Megachilidae	3	3.05	Long	Non-parasitic	Solitary	Cavity
Lithurgus cornutus	Megachilidae	4	4.09	Long	Non-parasitic	Solitary	Cavity
Megachile analis	Megachilidae	5	2.62	Long	Non-parasitic	Solitary	Cavity

Taxa	Family	Number of specimens	Inter-Tegular Distance (mm)	Tongue lenght	Parasitsm	Social status	Nesting
Megachile centuncularis	Megachilidae	20	2.72	Long	Non-parasitic	Solitary	Cavity
Megachile circumcincta	Megachilidae	8	2.82	Long	Non-parasitic	Solitary	Cavity
Megachile dorsalis	Megachilidae	2	2.63	Long	Non-parasitic	Solitary	Cavity
Megachile ericetorum	Megachilidae	15	3.39	Long	Non-parasitic	Solitary	Cavity
Megachile maritima	Megachilidae	1	NA	Long	Non-parasitic	Solitary	Soil
Megachile melanopyga	Megachilidae	3	3.03	Long	Non-parasitic	Solitary	NA
Megachile nigriventris	Megachilidae	5	3.55	Long	Non-parasitic	Solitary	Cavity
Megachile parietina	Megachilidae	1	3.87	Long	Non-parasitic	Solitary	Cavity
Megachile pilidens	Megachilidae	11	2.67	Long	Non-parasitic	Solitary	Cavity
Megachile pyrenaea	Megachilidae	12	1.63	Long	Non-parasitic	Solitary	Cavity
Megachile rotundata	Megachilidae	15	2.32	Long	Non-parasitic	Solitary	Cavity
Megachile versicolor	Megachilidae	1	3.14	Long	Non-parasitic	Solitary	Cavity
Megachile willughbiella	Megachilidae	28	3.59	Long	Non-parasitic	Solitary	Cavity
Melecta albifrons	Apidae	7	3.23	Long	Parasitic	-	-
Melecta luctuosa	Apidae	1	1.25	Long	Parasitic	-	-
Melitta leporina	Melittidae	27	2.39	Short	Non-parasitic	Solitary	Soil
Melitta nigricans	Melittidae	2	NA	Short	Non-parasitic	Solitary	Soil
Nomada alboguttata	Apidae	1	1.70	Long	Parasitic	-	-
Nomada atroscutellaris	Apidae	4	1.00	Long	Parasitic	-	-
Nomada bifasciata	Apidae	6	1.96	Long	Parasitic	-	-
Nomada distinguenda	Apidae	1	1.05	Long	Parasitic	-	-
Nomada fabriciana	Apidae	18	1.58	Long	Parasitic	-	-
Nomada ferruginata	Apidae	9	1.11	Long	Parasitic	-	-
Nomada flava	Apidae	10	1.99	Long	Parasitic	-	-
Nomada flavoguttata	Apidae	72	1.16	Long	Parasitic	-	-
Nomada flavopicta	Apidae	3	1.57	Long	Parasitic	-	-
Nomada fucata	Apidae	2	1.88	Long	Parasitic	-	-
Nomada goodeniana	Apidae	6	2.24	Long	Parasitic	-	-
Nomada guttulata	Apidae	2	1.21	Long	Parasitic	-	-
Nomada lathburiana	Apidae	30	2.18	Long	Parasitic	-	-
Nomada panzeri	Apidae	1	1.64	Long	Parasitic	-	-
Nomada ruficornis	Apidae	2	1.81	Long	Parasitic	-	-
Nomada succincta	Apidae	2	2.18	Long	Parasitic	-	-
Nomada tridentirostris	Apidae	1	1.15	Long	Parasitic	-	-
Nomada zonata	Apidae	11	1.67	Long	Parasitic	-	-
Nomioides minutissimus	Halictidae	8	0.80	Short	Non-parasitic	Solitary	Soil
Osmia aurulenta	Megachilidae	4	2.88	Long	Non-parasitic	Solitary	Cavity
Osmia bicolor	Megachilidae	2	2.70	Long	Non-parasitic	Solitary	Cavity
Osmia bicornis	Megachilidae	97	3.51	Long	Non-parasitic	Solitary	Cavity
Osmia brevicornis	Megachilidae	6	2.18	Long	Non-parasitic	Solitary	Cavity
Osmia caerulescens	Megachilidae	27	2.35	Long	Non-parasitic	Solitary	Cavity
Osmia cornuta	Megachilidae	194	3.36	Long	Non-parasitic	Solitary	Cavity
Osmia dives	Megachilidae	1	NA	Long	Non-parasitic	Solitary	Cavity
Osmia ferruginea	Megachilidae	1	NA	Long	Non-parasitic	NA	NA
Osmia gallarum	Megachilidae	2	NA	Long	Non-parasitic	Solitary	Cavity
Osmia inermis	Megachilidae	10	3.12	Long	Non-parasitic	Social	Cavity
Osmia leaiana	Megachilidae	2	2.67	Long	Non-parasitic	Solitary	Cavity
Osmia melanogaster	Megachilidae	13	2.84	Long	Non-parasitic	Solitary	Cavity
Osmia niveata	Megachilidae	14	2.46	Long	Non-parasitic	Solitary	Cavity
Osmia rufohirta	Megachilidae	3	2.53	Long	Non-parasitic	Solitary	Cavity
Osmia spinulosa	Megachilidae	1	2.03	Long	Non-parasitic	Solitary	Cavity
Osmia submicans	Megachilidae	12	2.16	Long	Non-parasitic	Solitary	Cavity
Panurgus dentipes	Andrenidae	58	1.67	Short	Non-parasitic	Solitary	Soil
Pseudapis diversipes	Halictidae	4	1.78	Short	Non-parasitic	NA	Soil
Sphecodes albilabris	Halictidae	2	2.18	Short	Parasitic	-	-
Sphecodes croaticus	Halictidae	3	1.18	Short	Parasitic	-	-
Sphecodes ephippius	Halictidae	9	1.45	Short	Parasitic	-	-
Sphecodes ferruginatus	Halictidae	6	1.42	Short	Parasitic	-	-
Sphecodes gibbus	Halictidae	2	1.71	Short	Parasitic	-	-
Sphecodes longulus	Halictidae	14	0.95	Short	Parasitic	-	-
Sphecodes majalis	Halictidae	2	1.29	Short	Parasitic	-	-
Sphecodes miniatus	Halictidae	1	0.99	Short	Parasitic	-	-
Sphecodes monilicornis	Halictidae	57	1.48	Short	Parasitic	-	-
Sphecodes niger	Halictidae	2	1.00	Short	Parasitic	-	-
Sphecodes pellucidus	Halictidae	1	0.36	Short	Parasitic	-	-
Sphecodes puncticeps	Halictidae	1	1.08	Short	Parasitic	-	-
Sphecodes reticulatus	Halictidae	2	1.21	Short	Parasitic	-	-
Sphecodes rufiventris	Halictidae	2	1.22	Short	Parasitic	-	-
Sphecodes scabricollis	Halictidae	3	0.27	Short	Parasitic	-	-
Stelis minuta	Megachilidae	1	1.34	Long	Parasitic	-	-
Stelis punctulatissima	Megachilidae	20	2.59	Long	Parasitic	-	-

Taxa	Family	Number of specimens	Inter-Tegular Distance (mm)	Tongue lenght	Parasitsm	Social status	Nesting
Tetralonia malvae	Apidae	257	2.61	Long	Non-parasitic	Solitary	Soil
Tetraloniella alticincta	Apidae	1	1.04	Long	Non-parasitic	Solitary	Soil
Tetraloniella dentata	Apidae	3	1.46	Long	Non-parasitic	Solitary	Soil
Thyreus ramosus	Apidae	1	2.40	Long	Parasitic	-	-
Thyreus truncatus	Apidae	1	NA	Long	Parasitic	-	-
Triepeolus tristis	Apidae	2	NA	Long	Parasitic	-	-
Xylocopa iris	Apidae	1	4.33	Long	Non-parasitic	Solitary	Cavity
Xylocopa valga	Apidae	13	6.59	Long	Non-parasitic	Solitary	Cavity
Xylocopa violacea	Apidae	30	6.54	Long	Non-parasitic	Solitary	Cavity

NA= none avaible
