# Supplementary material for: Decreasing Abundance, Increasing Diversity and Changing Structure of the Wild Bee Community (Hymenoptera: Anthophila) along an Urbanization Gradient
Source: PLoS One. 2014 Aug 13;9(8):e104679. doi: 10.1371/journal.pone.0104679 (PMC4131891; doi:10.1371/journal.pone.0104679)
Supplement: Table S3 — Significant correlation between landscape variables. (PDF) [file pone.0104679.s004.pdf]

Table S3: Significant correlation between landscape variables.

Radius = 500 m

	Impervious surface	Agricultural land	Wooded area	Open area	Connectivity of wooded area
Agricultural land	-0.75				
Wooded area	-0.45	-			
Open area	-	-	-		
Connectivity of wooded area	-	-	0.74	-	
Connectivity of open area	-	-	-	-	-

Radius = 1 000 m

	Impervious surface	Agricultural land	Wooded area	Open area	Connectivity of wooded area
Agricultural land	-0.81				
Wooded area	-0.5	-			
Open area	-	-	-		
Connectivity of wooded area	-	-	0.64	-	
Connectivity of open area	-	-	-		

Radius = 2 000 m

	Impervious surface	Agricultural land	Wooded area	Open area	Connectivity of wooded area
Agricultural land	-0.82				
Wooded area	-0.47	-			
Open area	-	-	-		
Connectivity of wooded area	-	-	0.5	-	
Connectivity of open area	-	-	-	0.71	-
